# Metabolic control of luteinizing hormone-responsive ovarian steroidogenesis

**DOI:** 10.1016/j.jbc.2024.108042

**Published:** 2024-11-29

**Authors:** Emilia Przygrodzka, Fatema Bhinderwala, Robert Powers, Renee M. McFee, Andrea S. Cupp, Jennifer R. Wood, John S. Davis

**Affiliations:** 1 Olson Center for Women's Health, Department of Obstetrics and Gynecology, University of Nebraska Medical Center, Omaha, Nebraska, USA; 2 Department of Chemistry, University of Nebraska-Lincoln, Lincoln, Nebraska, USA; 3 Nebraska Center for Integrated Biomolecular Communication, University of Nebraska-Lincoln, Lincoln, Nebraska, USA; 4 School of Veterinary Medicine and Biomedical Sciences, University of Nebraska-Lincoln, Lincoln, Nebraska, USA; 5 Department of Animal Science, University of Nebraska-Lincoln, Lincoln, Nebraska, USA; 6 VA Nebraska-Western Iowa Health Care System, Omaha, Nebraska, USA

**Keywords:** cyclic AMP, lipogenesis, progesterone, metabolism, ovary, protein kinase A (PKA), G protein-coupled receptor (GPCR)

## Abstract

The pituitary gonadotropin luteinizing hormone (LH) is the primary stimulus for ovulation, luteal formation, and progesterone synthesis, regardless of species. Despite increased awareness of intracellular signaling events initiating the massive production of progesterone during the reproductive cycle and pregnancy, critical gaps exist in our knowledge of the metabolic and lipidomic pathways required for initiating and maintaining luteal progesterone synthesis. Using untargeted metabolomics and metabolic flux analysis in primary steroidogenic luteal cells, evidence is provided for rapid LHCGR-stimulation of metabolic pathways leading to increased glycolysis and oxygen consumption. Treatment with LH stimulated posttranslational modifications of enzymes involved in *de novo* lipogenesis. Mechanistic studies implicated a crucial role for *de novo* fatty acid synthesis and fatty acid oxidation in energy homeostasis, LHCGR/PKA signaling, and, consequently, progesterone production. These findings reveal novel hormone-sensitive metabolic pathways essential for maintaining LHCGR/PKA signaling and steroidogenesis. Understanding hormonal control of metabolic pathways in steroidogenic cells may help elucidate approaches for improving ovarian function and successful reproduction or identifying metabolic targets for developing nonhormonal contraceptives.

The mammalian ovary has two principal functions: the production and release of mature ova capable of fertilization, and the synthesis and secretion of steroids, which are critical to ensure ovulation and pregnancy, but additionally to impact secondary sex characteristics, as well as bone, brain, immune, and cardiovascular health and metabolism (1, 2, 3). Ovarian follicles consisting of theca and granulosa cells provide a developmental niche allowing appropriate maturation of ova (4, 5). Mature ovarian follicles rupture during ovulation releasing ova into the fallopian tube, while the remnant of the follicle transforms into the corpus luteum, a highly vascularized, progesterone secreting powerhouse (3, 6). Granulosa and theca cells of the ruptured ovarian follicle transform into small and large luteal cells, respectively. Luteal cells are classified based on their size, morphological, and functional differences (7, 8, 9). Compared with the large luteal cells, the small luteal cells of the bovine corpus luteum are highly responsive to luteinizing hormone (LH) with greater increases in progesterone secretion (8). Progesterone, a steroid hormone produced by this transient endocrine gland is essential for ovulation (10), transport of ova through the fallopian tube (11), embryonic development, establishment and maintenance of the uterine environment to initiate and sustain pregnancy, as well as development of mothering behavior (7, 8). Recently, it was suggested that presence of the corpus luteum, which produces peptide hormones and vasoactive factors in addition to progesterone, reduces the risk of preeclampsia, the most common pregnancy complication in *in vitro* fertilization patients (9). If fertilization does not occur, the corpus luteum regresses and the reduction in progesterone allows for the progression of a new menstrual or estrous cycle (3, 12). Inadequate progesterone production at critical periods can lead to premature pregnancy loss in women and domestic animals (13). Given the importance of progesterone to successful reproduction and increasing infertility rates, studies to understand the hormonal and cellular control of progesterone offer insight into fertility regulation.

Among various luteotrophic factors, LH, a gonadotropin released by the pituitary gland, plays a crucial role in the formation, development, and maintenance of the corpus luteum in women and mammalian animals (3, 6). LH binds to its cognate receptor (LHCGR) present on the surface of luteal cells and rapidly elevates the production of cAMP, which consequently activates protein kinase A (PKA) (14, 15, 16). At the time of ovulation, this cascade of events recruits additional signaling events that in combination alter the phosphorylation of transcription factors, enhancing the expression of genes associated with angiogenesis, ovulation, cellular differentiation, steroidogenesis, and cell survival (17, 18, 19, 20). These events are also responsible for the posttranscriptional regulation of proteins involved in the maintenance of luteal function, such as enhanced lipolysis required to sustain cholesterol supply for progesterone synthesis or changes in mitochondrial dynamics (21, 22). Considerable effort has been placed in the past five decades to understand how gonadotropins control ovarian gene transcription and posttranslational modifications of various signaling proteins that positively or negatively impact transcription (21, 22, 23, 24). The focus of previous studies was to study the role of fatty acids crucial for granulosa cell or oocyte function (25). Little is known regarding the vital metabolic events driven by LH that contribute to lipid synthesis and progesterone secretion by the corpus luteum.

The current understanding of the metabolic phenotype of luteal cells is limited and largely based on the luteal transcriptome and proteome (18, 26). Recently, we identified changes in the expression of genes encoding proteins associated with the metabolism of glucose and lipids in luteal cells in comparison to ovarian follicle cells (18, 27). However, significant gaps in our knowledge exist concerning the metabolic pathways governing the trophic actions of LH on steroidogenesis and maintenance of luteal function. Previous investigations targeted a limited number of specific metabolites such as glucose, lactate, or pyruvate in the whole ovary, granulosa cells, or luteinized tissue (28, 29, 30, 31, 32, 33). However, there are no studies providing information on acute global metabolic changes induced by gonadotropin stimulus in the highly steroidogenic luteal cells. Metabolic profiling of steroidogenic luteal cells can provide insight into the cellular metabolic pathways that support the pronounced increase in steroidogenesis observed following ovulation, which is required for the establishment and maintenance of pregnancy. Herein, using two unbiased metabolic approaches, *i.e.*, mass spectrometry and NMR, we characterized the temporal metabolic changes in highly LH-responsive primary steroidogenic luteal cells. Further biochemical analysis determined the effects of LH on glucose uptake, glycolysis, and mitochondrial respiration. We also evaluated the flux of glucose carbons in different metabolic pathways and assessed the significance of specific LH-mediated metabolic pathways controlling steroidogenesis using selective small molecule inhibitors. Mechanistic *in vitro* experiments identified acetyl-CoA carboxylase alpha (ACACA) and ATP citrate lyase (ACLY), two enzymes involved in *de novo* lipogenesis, as targets for LH-stimulated PKA signaling and disruption of ACLY phosphorylation or enzyme activity as well as transport of fatty acids to mitochondria acutely blocked LHCGR/PKA signaling and LH-stimulated progesterone synthesis. Notably, this study was conducted with LH-responsive primary steroidogenic small luteal cells from the bovine corpus luteum, which is a powerful model for studying human ovarian physiology (34). Like women, cows are mono-ovulatory with multiple waves of follicular development, and the length of the luteal phase and pregnancy are similar (34). Moreover, the signaling pathways triggered by LHCGR are evolutionarily conserved (3) and LH is necessary for the formation and maintenance of the corpus luteum, and progesterone is required to establish and maintain pregnancy in both species (34).

## Results

### LH induces acute metabolic alterations and cholesterol utilization in steroidogenic luteal cells

**Figure 1:**
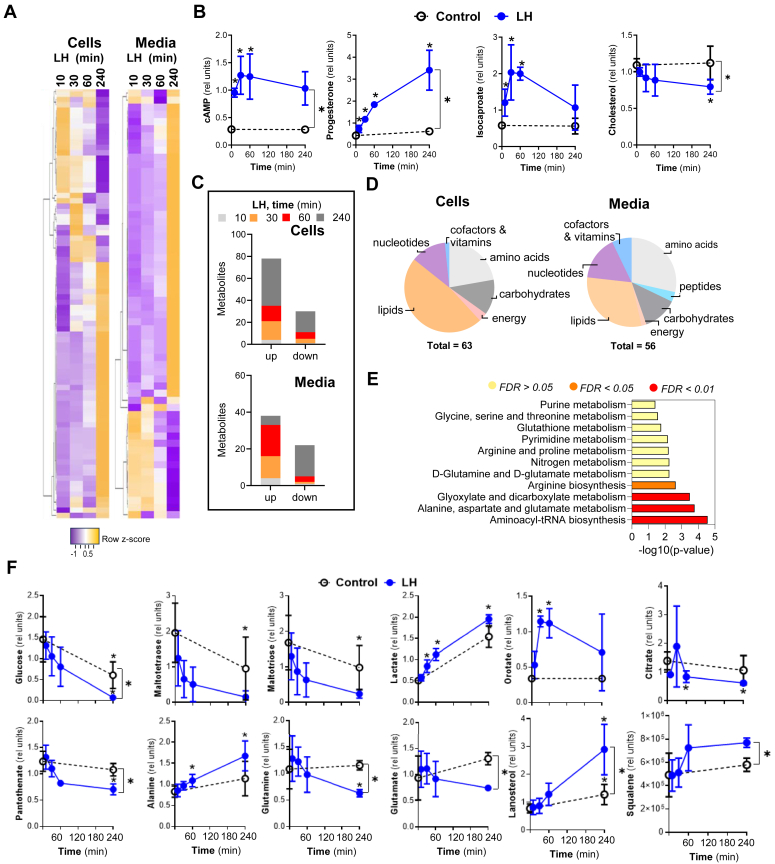
**Luteinizing hormone (LH) stimulates acute changes in cellular lipids and glucose metabolism in luteal cells.***A*, heatmaps showing the most significant (≥1.5 or ≤ −1.5) time-dependent changes post-LH treatment in media and cell extracts. The heatmap was prepared using http://www.heatmapper.ca/. *B*, changes in cellular or media content of cyclic AMP (cAMP), progesterone, isocaproate, and cholesterol in small luteal cells treated with LH (10 ng/ml) for 0-, 10-, 30-, 60-, and 240-min. Values are presented as relative units (RU) and means ± SD, n = 3. *C* and *D*, time-dependent changes in metabolite concentrations and classes in media and cell extracts. *E*, metabolic pathways changed post-LH treatment. Analysis was done using MetaboAnalyst (Version 3.0, URL: http://www.metaboanalyst.ca) and the most significantly changed (≥1.5 or ≤ −1.5) metabolites in media and cell extracts. False discovery rate (FDR) presented with different colors. *F*, changes in cellular or media content of selected metabolites in the small luteal cells treated with LH (10 ng/ml) for 0 to 240-min. Values are presented as relative units (RU) and means ± SD, n = 3. For all graphs, ∗ indicates *p* < 0.05 as determined by the *t* test.

This study was designed to determine the rapid action of LH on acute metabolic changes in steroidogenic bovine small luteal cells. To accomplish this, an untargeted approach was used to measure metabolites in the whole cell extracts and conditioned medium after incubation of freshly isolated luteal cell suspensions with LH (10 ng/ml) for 10, 30, 60, and 240 min (Fig. 1, *A*–*F*; Table S1). This is a physiologically relevant LH concentration that provides maximal steroid responses *in vitro* (21, 23, 24, 35). Because luteal cells continuously produce progesterone, control samples were collected at times 0 and 240 min to determine the temporal changes associated with the basal production of progesterone.

Mass spectrometry revealed acute and prominent changes in metabolite concentrations depending on treatment and time in both cell extracts and media (Fig. 1*A*; Table S1). As expected, LH rapidly increased cAMP in cells (4.4-fold) after 10 min of incubation, with maximal levels attained within 30 to 60 min (Fig. 1*B*). A corresponding rapid and continual increase in progesterone secretion was observed. LH also stimulated an acute elevation (3.5-fold) in isocaproate, a product of cholesterol cleaved by CYP11A1, the mitochondrial cholesterol side-chain cleavage enzyme, with a concomitant reduction (28% reduction) in total cellular cholesterol content after 240 min (Fig. 1*B*). These findings confirm that small steroidogenic luteal cells used in these studies provide an appropriate model to examine metabolic events activated during the steroidogenic response to LH.

Treatment with LH rapidly altered the luteal cell metabolic profile. Briefly, 276 and 117 metabolites were identified in cell extracts and conditioned culture media, respectively. Incubation with LH for 10 min induced significant changes (*p* < 0.05) in five metabolites in cell extracts and media samples; after 30 and 60 min, the number of metabolites increased to 20, and after 240 min of treatment, it increased to 34. In control samples, 54 metabolites showed significant changes during the 240 min incubation (Fig. 1*C*; Table S1). The most significantly changed metabolites in cells and media were categorized as lipids, amino acids, nucleotides, and carbohydrates (Fig. 1*D*). Enrichment analysis indicated significant changes (*p* < 0.05; false discovery rate < 0.05) in aminoacyl-tRNA biosynthesis; alanine, aspartate, and glutamate metabolism; glyoxylate and dicarboxylate metabolism and arginine biosynthesis (Fig. 1*E*; Table S1).

### LH enhances metabolic pathways leading to lipid synthesis and pathways regulating energy and redox homeostasis in luteal cells

Glucose is a primary energetic substrate in each living cell (36). Once inside the cells, glucose molecules are processed to produce pyruvate and lactate. Further, pyruvate can be transported to the mitochondria *via* mitochondrial pyruvate carriers, where it is utilized in the TCA cycle to produce energy and intermediates required for *de novo* lipogenesis or nucleotide synthesis (36).

LH induced a substantial decrease in the intracellular concentration of glucose (59% reduction), as well as the glucose storage compounds—maltotriose and maltotetraose (Fig. 1*F*), polysaccharides with glucose molecules linked with α-1,4 glycosidic bonds (86% and 92% reduction). In media samples, a dramatic drop in fructose and glucose concentrations (90% and 51% reduction) was found after 30 min with LH treatment (Table S1). The LH-stimulated drop in glucose was associated with a 1.3-fold increase in lactate in media samples, evidence of enhanced glycolysis (Fig. 1*F*). Glucose can also be directed to the pentose phosphate pathway (PPP) and hexosamine biosynthetic pathway (HBP), which leads to the synthesis of nucleotides or reduced NADPH and glycolipids, and glycoproteins, respectively (37). LH treatment elevated levels of orotate, a pyrimidine precursor, reaching maximal levels within 30 to 60 min (3.4-fold) in cells, indicating enhanced activity of the PPP in response to LH (Fig. 1*F*). A rapid utilization of citrate (56% reduction) was observed in cell extracts suggesting increased activity of the TCA cycle (Fig. 1*F*). In addition, at 240 min, LH provoked a depletion (35%) of pantothenate, a precursor of coenzyme A (CoA), which is a cofactor required for acetyl-CoA formation and lipid metabolism (Fig. 1*F*) (38). Herein, under serum-free culture conditions, lanosterol and squalene, precursors for the *de novo* synthesis of cholesterol, were elevated (2.5- and 1.5-fold) in cells and media 240 min after LH treatment, suggesting enhanced activity of *de novo* lipogenesis and enzymes involved in cholesterol synthesis (Fig. 1*F*).

LH raised the concentration of alanine (Fig. 1*F*) and tryptophan (Table S1), two amino acids that can be used as a source of pyruvate for the TCA cycle. Beyond pyruvate, the TCA cycle can be replenished by anaplerosis by conversion of glutamine and glutamate to α-ketoglutarate (37). Glutamine and glutamate were significantly reduced (56% and 53%) in media samples 240 min after LH treatment, indicating the possible use of these amino acids to generate TCA intermediates or glutathione, a known antioxidant (39), especially in view of the rapid and persistent decreased levels of cysteine-glutathione-disulfide (CYSH-GSH; 33% reduction) in cells following LH treatment.

### LH stimulates hydrolysis of phospholipids

Phospholipids, which are abundant in cell membranes, can be hydrolyzed by phospholipase A (PLA) to release fatty acids, resulting in the formation of lysophospholipids. Fatty acids can be used as substrates for mitochondrial energy production or synthesis of lipid mediators, including prostanoids, leukotrienes, or steroids (38). Substantial increases in intracellular concentrations of various lysophospholipids at 240 min of LH treatment suggest a delayed enhanced phospholipase A2 (PLA2) activity after gonadotropin stimulus (Table S1). Enhanced PLA activity in LH-treated luteal cells is consistent with previous reports of elevated cytosolic PLA2 levels and activity in granulosa cells of primate periovulatory follicles stimulated with human chorionic gonadotropin (40).

Results obtained by mass spectrometry analysis were confirmed by NMR spectroscopy (SI Appendix, Fig. S1, *A* and *B*), where metabolic changes were analyzed in cell extracts obtained from suspensions of small luteal cells incubated with LH for 240 min. Augmented concentrations of glycine, alanine, branched-chain amino acids, threonine, and lactate were identified. Simultaneously, depletions were observed in metabolites related to the TCA cycle and redox homeostasis, such as glutamate and glutamine, citrate, aspartate, arginine, glutathione, and choline-containing compounds following LH treatment (SI Appendix, Fig. S1*B*).

### LH stimulates glycolysis and mitochondrial respiration

**Figure 2:**
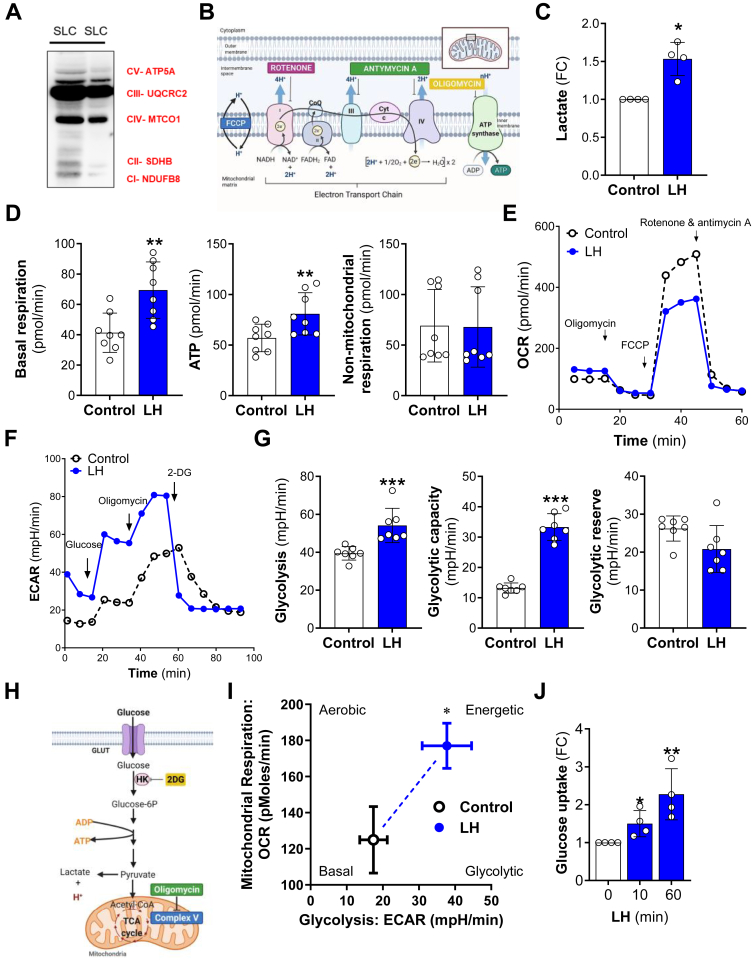
**LH affects mitochondrial respiration and glycolysis.***A*, representative Western blot using a total OXPHOS antibody cocktail showing content of proteins of the electron transport chain in the small luteal cells. *B*, diagram presenting electron transport chain and function of inhibitors used in seahorse analysis. Mitochondrial respiration was measured as oxygen consumption rate (OCR) following a sequential addition of inhibitors of mitochondrial function: oligomycin, carbonyl cyanide-p-trifluoromethoxyphenylhydrazone (FCCP), and a combination of rotenone and antimycin A. *C*, lactate concentration measured in medium post seahorse analysis. Data are presented as a fold change (FC) and mean ± SD (n = 4). *D*, basal respiration, ATP production, and nonmitochondrial respiration in untreated (control) and LH-treated (60 min) luteal cells. Data are presented as mean ± SD (n = 3 with 2–3 technical replicate). *E*, representative graph showing oxygen consumption rate (OCR) in the small luteal cells treated with LH (10 ng/ml) for 60 min (*blue solid line*) and control cells (*black dotted line*). *F*, representative graph showing changes in extracellular acidification rate (ECAR) in the small luteal cells untreated (control) or treated with LH (10 ng/ml) for 60 min. *G*, glycolysis, glycolytic capacity, and glycolytic reserve in control and LH-treated (60 min) cells. Data are represented as mean ± SD (n = 3 with 2–3 technical replicate). *H*, diagram explaining site of action of inhibitors used in seahorse analysis. Glycolytic rate was analyzed using seahorse glycolytic rate assay. Glycolytic rate was measured as ECAR following a sequential addition of glucose, oligomycin (inhibitor of ATP synthase), and 2-deoxyglucose (2-DG; inhibitor of hexokinase- HK). *I*, bioenergetics phenotype of untreated (control) and LH-treated cells done basing on OCR and extracellular acidification rate (ECAR) values obtained from seahorse analysis (n = 3). *J*, glucose uptake assay done using luminescent method for untreated (control) and LH-treated cells for 10- and 60-min. Data are presented as a fold change (FC) and mean ± SD (n = 4). For all graphs ∗, ∗∗, and ∗∗∗, and mean significant change with *p* < 0.05, *p* < 0.01, and *p* < 0.001, respectively, as determined by *t* test or one-way ANOVA followed by Bonferroni *post hoc* test. LH, luteinizing hormone; OCR, oxygen consumption rate; OXPHOS, oxidative phosphorylation.

Cells produce energy through two major pathways: oxidative phosphorylation (OXPHOS) and glycolysis. During oxidation and reduction reactions, substrates such as pyruvate, glutamine, and fatty acids are converted within the mitochondria into ATP (36, 37). Due to the observed changes in the concentration of glucose and glutamine, we next determined the effect of LH on mitochondrial respiration. The components of the electron transport chain (ETC) in small luteal cells were analyzed using a cocktail of antibodies specific for determining the relative levels of OXPHOS complexes in mitochondria. All five complexes (CI-CV) were detected (Fig. 2*A*). Mitochondrial respiration was measured in real-time as oxygen consumption rate (OCR) in intact primary small luteal cells using the seahorse mito stress assay during which OCR is measured before (basal respiration) and after injections with inhibitors of the ETC (Fig. 2*B*). Along with elevated concentrations of lactate (1.5- fold) (Figs. 1*F* and 2*C*), treatment with LH increased basal respiration and ATP production (1.7-, and 2.8- fold) compared to control cells. No changes were observed in nonmitochondrial respiration (Fig. 2, *D* and *E*). Maximal respiratory capacity measured after 2-(2-(4-(trifluoromethoxy) phenyl) hydrazinylidene)-propanedinitrile) (FCCP) injection was markedly lower (28% reduction) in cells incubated with LH *versus* control cells, indicating a reduced availability of substrates used for oxidation following treatment with LH (Fig. 2*E*). Furthermore, an elevated extracellular acidification rate (ECAR) was found in LH-treated cells (SI Appendix, Fig. S2*A*).

Our mass spectrometry data and previous studies indicate that LH affects glucose uptake and glycolysis (28, 33, 41, 42). Thus, further analysis of glycolytic rate and glucose uptake was assessed in LH-treated small luteal cells. Seahorse analysis using the glycolysis stress assay revealed elevated ECAR in LH-treated cells compared to control cells (Fig. 2*F*). Glucose and oligomycin injection increased ECAR in LH-treated cells *versus* control, reflecting enhanced glycolysis and glycolytic capacity after gonadotropin stimulus (1.5 and 3-fold), while the glycolytic reserve remained unchanged (Fig. 2, *F*–*H*). Elevated ECAR and OCR values indicate that LH acutely promotes an energetic phenotype in luteal cells (Fig. 2*I*), which signifies that LH stimulates metabolic pathways that boost the cellular flow of metabolites for both glycolysis and mitochondrial respiration in luteal cells. A bioluminescent method based on detecting 2-deoxyglucose-6-phosphate revealed that LH stimulated significant increases in glucose uptake within 10 min, and uptake continued to rise over 60 min (2.2-fold; Fig. 2*J*).

Because our previous microarray studies (GSE83524) revealed transcripts for glucose transporters (*GLUT 1*, *3*, *8*, and *13*) in luteal cells, with *GLUT1* being the most highly expressed (Fig. S2*B*), we measured glucose uptake in cells pretreated for 60 min with a selective inhibitor of GLUT1 (BAY876) or an inhibitor of GLUT1 and GLUT4 (Fasentin). Treatment with both GLUT inhibitors abolished the stimulatory effect of LH on glucose uptake, indicating the importance of GLUT1 in LH-responsive glucose uptake in luteal cells (SI Appendix, Fig. S2*C*); however, they did not prevent LH-stimulated progesterone synthesis (SI Appendix, Fig. S2*D*). Also, inhibition of hexokinase, the enzyme catalyzing the first essential step of glucose metabolism, *i.e.*, conversion of glucose into glucose-6-phosphate (43), using Lonidamine, a selective hexokinase inhibitor, did not affect steroidogenesis in cells treated with LH (SI Appendix, Fig. S2*E*).

### LH directs glucose flux through glycolysis, pentose phosphate pathway, and hexosamine biosynthetic pathway

**Figure 3:**
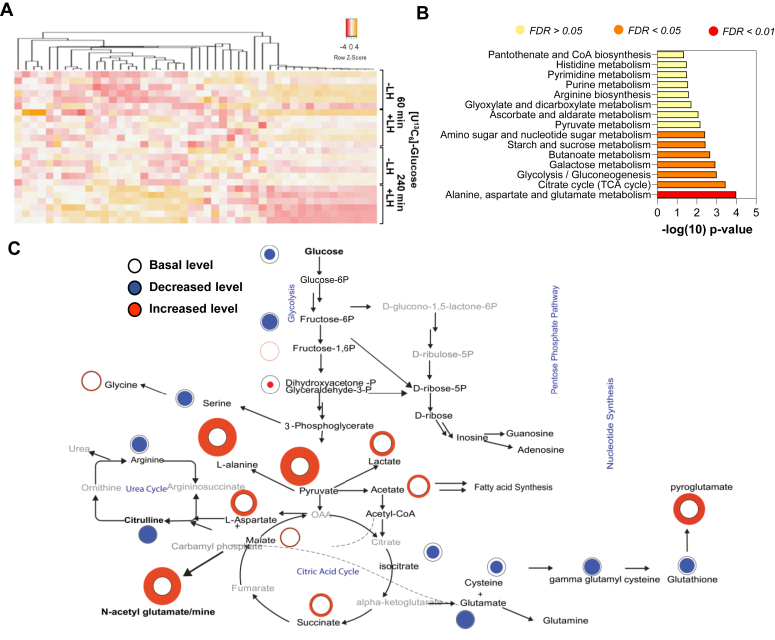
**LH stimulates glucose metabolism in luteal cells.** Enriched preparation of small luteal cells treated with [U^13^C_6_]-glucose (5 mM) alone or in the presence of LH (10 ng/ml) for 60- and 240-min. Cellular metabolites were analyzed by Nuclear Magnetic Resonance (NMR) spectroscopy. *A*, heatmap representing the most significant changes in [U^13^C_6_]-labeled metabolites. *B*, enrichment pathway analysis done using MetaboAnalyst (Version 3.0, URL: http://www.metaboanalyst.ca). Analysis was performed using the most significantly (*p* < 0.05) changed metabolites in cells and media. *C*, flow chart showing the most significant changes and metabolic pathways in cell extracts cultured in the presence of LH. *Black*, *blue*, and *red circles* represent the basic, decreased, or increased concentration of metabolites. LH, luteinizing hormone.

Next, we performed a fluxomics analysis to determine how small luteal cells use glucose after gonadotropin stimulus. Freshly isolated suspensions of luteal cells were incubated in media containing 5 mM [U^13^C_6_]-glucose and then treated with or without LH for 60 or 240 min. We discerned the time-dependent effects of LH on metabolic changes by using NMR (Fig. 3, *A* and *B*). Incubation with LH rapidly depleted [U^13^C_6_]-glucose from cells (48% reduction) and media (52% reduction) (Fig. 3, *C* and *D*) with a simultaneous increase (by 15–16-fold) in progesterone production (SI Appendix, Fig. S3*A*; Table S2). Bioinformatics analysis revealed the most significant (*p* < 0.05; false discovery rate < 0.05) enrichment of glucose metabolites in the following pathways: alanine, aspartate, and glutamate metabolism; TCA cycle; glycolysis/gluconeogenesis; galactose metabolism and butanoate metabolism (Fig. 3*B*) suggesting changes within pathways related with production of energy, synthesis of nucleotides and proteins or cofactors catalyzing synthesis of lipids and proper function of steroidogenic machinery (39).

A prominent decrease in metabolites related to the glycolytic pathway, such as glyceraldehyde-3-phosphate (38% depletion) and an increase in the production of lactate (1.5- fold increase) were observed in response to LH (Fig. 3*C*). Incubation with LH also induced acute changes within the PPP and the HBP reflected by elevated concentrations of uridine (3-fold), CTP/UTP (6.7-fold), and uridine diphosphate (UDP)-glucose (4-fold) (Fig. 3*C*; SI Appendix, Fig. S3*B*).

### LH leads glucose flow through the TCA cycle, lipid metabolism, and redox homeostasis

LH treatment enhanced the concentration of metabolites related with the TCA cycle, such as ^13^C-labeled-pyruvate and -succinate (1.5-fold increase for both), as well as decreased (34%) content of ^13^C-labeled-isocitric acid and malate (Fig. 3*C*; SI Appendix, Fig. S3*B*). There was also a significant increase in the content of NADH in LH-treated cells (3.1-fold; Fig. 3*C*; SI Appendix, Fig. S3*B*), which is produced during glycolysis and the TCA cycle indicating enhanced activity of these pathways (36, 39). There was also an elevated concentration of alanine and aspartate (2.2 and 1.3-fold), two amino acids used in the TCA cycle for gluconeogenesis, or nucleotide synthesis (39). Compared to control, LH decreased the content of carnitine (20% depletion), which transports long-chain fatty acids into mitochondria to be oxidized for ATP production, while increasing the content of CoA (1.3-fold) and acetate (1.3-fold) (Fig. 3; SI Appendix, Fig. S3*B*), suggesting enhanced activity in pathways supplying cells with acetyl-CoA. There were also changes in metabolites related to redox homeostasis, including an increase in glutathione (2.0-fold) concentration (Fig. 3*C*; SI Appendix, Fig. S3*B*). A summary of the flux of [U^13^C_6_]-glucose in response to LH is shown in Figure 3*C*.

### Pyruvate synthesis and TCA cycle activity are crucial for LH-stimulated steroidogenesis

**Figure 4:**
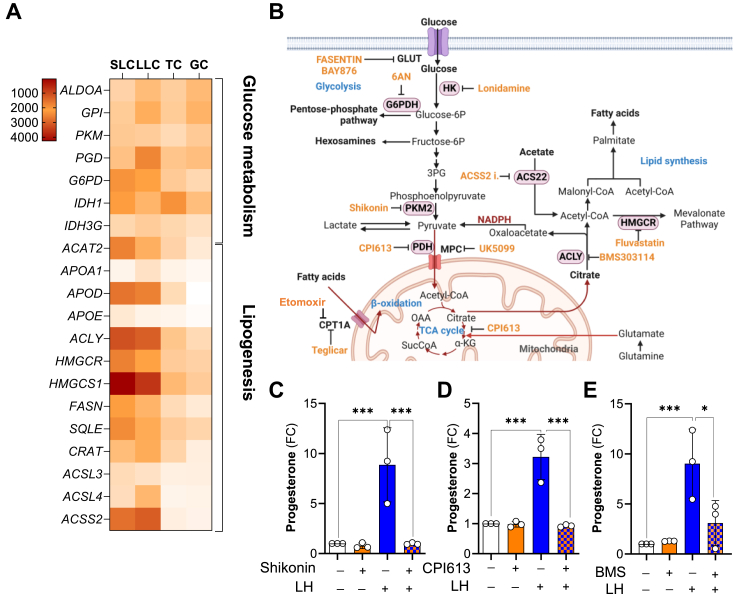
**Glycolysis, TCA cycle, and *de novo* synthesis of fatty acids are vital for steroidogenic capacity of small luteal cells.** Small luteal cells were pretreated with inhibitors of selected metabolic pathways for 60 min and then treated with LH (10 ng/ml) for 240 min. Progesterone concentration was measured in media samples. *A*, heatmap representing the expression of genes related to lipid metabolism in granulosa and theca cells (GC and TC) as well as small and large luteal cells (SLC and LLC). *B*, flow chart showing sequence of metabolic events occurring in cells with information of inhibited enzymes and applied inhibitors. *C*–*E*, progesterone production by small luteal cells pretreated with inhibitors [PKM2-Shikonin (1 μM), PDH and KDGH-CPI613 (25 μM), or ACLY-BMS30314 (25 μm)]. Data are presented as a fold change (FC) and mean ± SD (n = 3). ∗, ∗∗∗ mean significant change with *p* < 0.05, *p* < 0.001 as determined by one-way ANOVA followed by Bonferroni *post hoc* test. ACLY, ATP citrate lyase; LH, luteinizing hormone; PDH, pyruvate dehydrogenase; PKM2, pyruvate kinase 2.

Luteal cells are abundant in genes encoding factors related to cholesterol (*APOA1*, *APOD*, *APOE*, *HMGCR*, *HMGCS1*, *SQLE*, and *ACAT2*) and fatty acids synthesis or metabolism (*ACLY*, *ACSS2*, *FASN, CRAT, ACSL3*, and *ACSL4*) in comparison to theca and granulosa cells (GSE83524; Fig. 4*A*). The expression of genes encoding glucose transporters (SI Appendix, Fig. S2*B*) and glucose metabolism (*ALDOA*, *LDHB*, *G6PD*, *PGD, IDH1*, and *IDH3G*) were also elevated during the follicle-to-luteal transition (Fig. 4*A*).

Further, experiments were conducted to determine the potential role of metabolic pathways related to glucose and lipid metabolism on the steroidogenic response to LH (Fig. 4*B*). Because we observed LH-stimulated effects on glucose flow through the PPP, luteal cells were treated with 6-aminonicotinamide to block glucose-6-phosphate dehydrogenase (G6PD), an enzyme necessary for directing glucose to the PPP and responsible for NADPH production (39). Pretreatment with this inhibitor did not block LH-simulated progesterone synthesis despite applying relatively high concentrations of the compound (SI Appendix, Fig. S4*A*).

Next, we inhibited pyruvate kinase M2 (PKM2), catalyzing the last step in glycolysis, *i.e.*, dephosphorylation of phosphoenolpyruvate to generate pyruvate (Fig. 4*B*). Pretreatment with increasing concentrations of the PKM2 inhibitor-Shikonin decreased LH-stimulated progesterone production (Fig. 4*C*; SI Appendix, Fig. S4*B*). Treatment with Shikonin also reduced LH-stimulated ECAR, glycolysis, glycolytic capacity, and glycolytic reserve (SI Appendix, Fig. S4, *C* and *D*). Since luteal cells treated with LH showed prominent changes within the TCA cycle, we also used CPI-613, an inhibitor of pyruvate dehydrogenase and α-ketoglutarate dehydrogenase (44) catalyzing the conversion of pyruvate to acetyl-CoA and α-ketoglutarate to succinyl-CoA, respectively (Fig. 4*B*). Pretreatment with CPI-613 profoundly decreased progesterone production in cells treated with LH (Fig. 4*D*), suggesting a crucial role of glycolysis and pyruvate production, as well as the TCA cycle, in luteal steroidogenesis.

### Fatty acids are an important source of energy and fundamental for steroidogenesis in luteal cells

Our global metabolomics analysis identified a rapid depletion of citrate in luteal cells treated with LH. Citrate produced in the TCA cycle can be transported to the cytoplasm and cleaved to acetyl-CoA and oxaloacetate by ACLY with concomitant hydrolysis of ATP to ADP and phosphate. The generated oxaloacetate can be converted to malate and pyruvate with NADPH production catalyzed by malate enzyme. Acetyl-CoA can also be produced from acetate in the reaction catalyzed by acetyl-CoA synthetase short-chain family member 2 (ACSS2) (45). The product, acetyl-CoA, is a substrate for fatty acid and cholesterol synthesis (Fig. 4*B*). Genes encoding both ACLY and ACSS2 are abundant in luteal cells (Fig. 4*A*). Pretreatment for 60 min with a specific ACSS2 inhibitor prior to treating luteal cells with LH for 240 min did not change LH-stimulated progesterone production (SI Appendix, Fig. S5*A*). To determine whether cholesterol synthesis supports the acute steroidogenic response to LH, cells were treated with an inhibitor of 3-hydroxy-3-methylglutaryl-CoA reductase (HMGCR), an enzyme considered as the rate-limiting enzyme for this pathway (Fig. 4*B*). Incubation of luteal cells in the presence of fluvastatin, a clinically used inhibitor of HMGCR (46), did not affect LH-stimulated progesterone production, suggesting that fatty acid synthesis, instead of cholesterol synthesis, may have an essential role in the maintenance of steroidogenic capacity of luteal cells (SI Appendix, Fig. S5*B*). Thus, we focused our further experiments on the function of ACLY.

**Figure 5:**
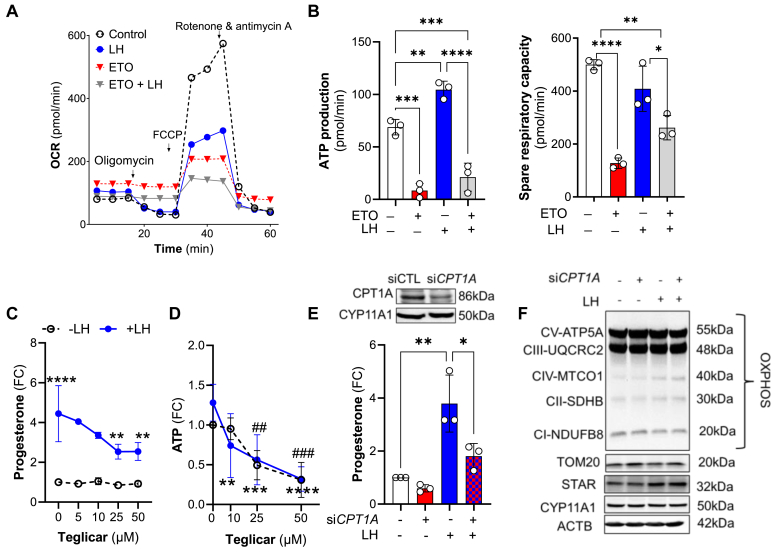
**Endogenous fatty acids are an essential source of energy in luteal cells.***A*, representative graph showing oxygen consumption rate (OCR) in the small luteal cells pretreated with etomoxir (ETO; 30 μM) and then treated with LH (10 ng/ml) for 60 min. *B*, ATP production and spare respiratory capacity in the luteal cells pretreated with etomoxir (ETO) and then treated with LH. Data are represented as mean ± SD (n = 3). *C*, dose-dependent effects of CPT1A inhibitor (Teglicar; 5–100 μM) on progesterone production by untreated (control) and LH-treated small luteal cells. Data are represented as fold change (FC) and mean ± SD (n = 3–5). *D*, ATP production by luteal cells pretreated with CPT1A inhibitor (Teglicar; 10–50 μM) and then treated with LH (10 ng/ml) for 240 min. Data are presented as fold change and mean ± SD (n = 3–5). *Asterisks* ∗∗, ∗∗∗, ∗∗∗∗ mean significant change with *p* < 0.05, *p* < 0.01, and *p* < 0.001, respectively for cells treated with LH alone *versus* Teglicar + LH. Symbols ^##^ and ^###^ represent significant change with *p* < 0.01 and *p* < 0.01, for cells treated with Teglicar *versus* control (untreated). *E*, progesterone production by small luteal cells with knockdown *CPT1A* (si*CPT1A*; 50 nM) and treated with LH (10 ng/ml) for 240 min. Control cells were transfected with siControl (50 nM). Data are presented as a fold change (FC) and mean ± SD (n = 3). Efficiency of siRNA transfection was confirmed using Western blotting and is presented above the bar graph. *F*, representative blots showing content of electron transport chain (ETC) proteins in the small luteal cells with knockdown *CPT1A* (si*CPT1A*; 50 nM) and then treated with LH (10 ng/ml) for 240 min. For all graphs (*A*–*C* and *E*) ∗, ∗∗, ∗∗∗, ∗∗∗∗ mean significant change with *p* < 0.05, *p* < 0.01, *p* < 0.001 and *p* < 0.0001. Data were analyzed by one- or two-way ANOVA followed by the Bonferroni *post hoc* test. CPT1A, carnitine palmitoyltransferase 1A; LH, luteinizing hormone; siRNA, silencing RNA.

Pretreatment with increasing concentrations of ACLY inhibitor, BMS303141 (BMS), caused concentration-dependent reductions in the stimulatory effect of LH on progesterone production in small luteal cells (20–65%), implicating *de novo* lipogenesis in the steroidogenic response (Fig. 4*E*; SI Appendix, Fig. S5*C*). Next, we performed experiments to determine whether mitochondria utilize endogenous fatty acids to generate ATP and if fatty acids are required for progesterone production in luteal cells. Pretreatment with etomoxir, an inhibitor of carnitine palmitoyltransferase 1A (CPT1A), which transports fatty acids into mitochondria (38) (Fig. 4*B*), significantly inhibited the stimulatory effects of LH on OCR (Fig. 5*A*), ATP production (Figs. 5*B*, 80% reduction), and progesterone synthesis (Fig. 5*C*), implicating fatty acids as important mediators of luteal function (Fig. 5, *A*–*C*; SI Appendix, Fig. S5*D*). This finding was confirmed by pretreatment with another inhibitor of CPT1A, Teglicar, where increasing concentrations of Teglicar decreased LH-stimulated progesterone (10–57% reduction) and ATP production (42–76% reduction) measured using luminescence (Fig. 5, *C* and *D*). Furthermore, genetic knockdown of *CPT1A* also inhibited LH-stimulated progesterone production (53% reduction) (Fig. 5*E*). Knockdown of CPT1A did not change the content of mitochondrial OXPHOS proteins and TOM20 or steroidogenic machinery proteins (STAR, CYP11A1) (Fig. 5*F*).

### ACLY is a target of LHCGR/PKA signaling and crucial for gonadotropin signal transduction

**Figure 6:**
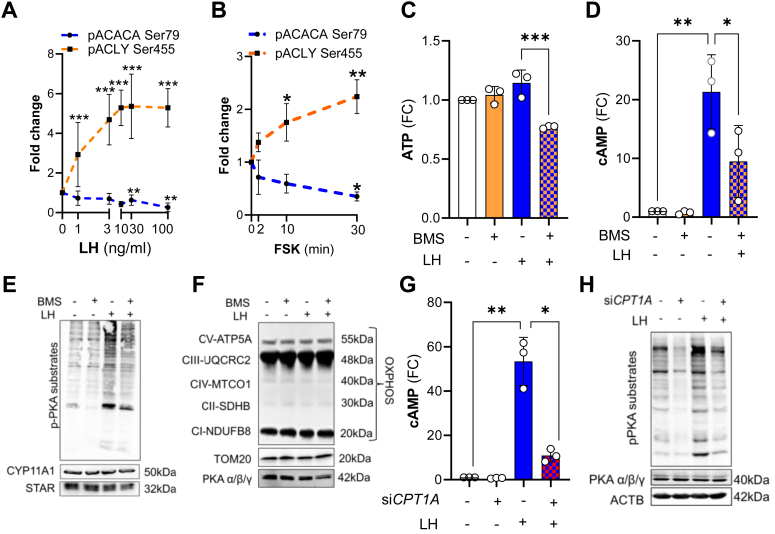
**LHCGR/PKA pathway triggers phosphorylation of enzymes involved in *de novo* lipogenesis.***A*, phosphorylation of ACC1 (ACACA) Ser79 and ACLY Ser455 in the small luteal cells treated with different concentrations of LH (1–100 ng/ml) for 30 min. Data are presented as fold change and mean ± SD (n = 3–5). *B*, phosphorylation of ACC1 Ser79 and ACLY Ser455 in the small luteal cells treated with cAMP/PKA activator-forskolin (FSK; 10 μM) for 2 to 30 min. Data are presented as a fold change (FC) and mean ± SD (n = 3). Data normalized to total protein loaded on each lane. *C*, ATP production in the small luteal cells pretreated with ACLY inhibitor (BMS303141; 10–50 μm) and then treated with LH (10 ng/ml) for 240 min. Data are presented as fold change (FC) and mean ± SD (n = 3). *D*, cyclic AMP (cAMP) production by the small luteal cells pretreated with ACLY inhibitor (BMS303141; 25 μm) and then treated with LH (10 ng/ml) for 240 min. Data are presented as fold change (FC) and mean ± SD (n = 3). *E* and *F*, representative blots showing phosphorylation of PKA substrates and content of steroidogenic proteins (STAR, CYP11A1), electron transport chain proteins or marker of mitochondria (TOM20) and PKA catalytic subunits in the small luteal cells pretreated with ACLY inhibitor (BMS303141; 25 μM) and then treated with LH (10 ng/ml) for 240 min. *G*, cyclic AMP (cAMP) production by the small luteal cells with knockdown CPT1A (si*CPT1A*; 50 nM) and then treated with LH (10 ng/ml) for 240 min. Data are presented as fold change (FC) and mean ± SD (n = 3). *H*, representative blots showing phosphorylation of PKA substrates and content of PKA catalytic subunits in the small luteal cells with knockdown CPT1A (si*CPT1A*; 50 nM) and then treated with LH (10 ng/ml) for 240 min. For all graphs ∗, ∗∗, ∗∗∗ and ∗∗∗∗ mean significant change with *p* < 0.05, *p* < 0.01, *p* < 0.001, and *p* < 0.0001. Data were analyzed by one-way ANOVA followed by the Bonferroni *post hoc* test. ACACA, acetyl-CoA carboxylase alpha; ACLY, ATP citrate lyase; CPT1A, carnitine palmitoyltransferase 1A; LH, luteinizing hormone; PKA, protein kinase A.

Considering the crucial role of fatty acids in LH-mediated stimulatory effects on progesterone by highly steroidogenic luteal cells, we tested the effects of LH on the phosphorylation of enzymes involved in fatty acid synthesis. PKA regulates the phosphorylation and activation state of at least two enzymes involved in *de novo* lipogenesis-ACACA and ACLY (38, 47). Small luteal cells were treated with increasing concentrations of LH or forskolin (FSK), an activator of adenylyl cyclase, for up to 30 min. LH and FSK significantly stimulated the phosphorylation of ACLY at Ser455, a site-specific for enzyme activation (48). Phosphorylation of ACLY increased 3-fold at the lowest concentration of LH (1 ng/ml), and the extent of phosphorylation continued to rise with higher concentrations of LH, reaching the maximal level (5.4-fold increase) after treatment with 10 ng/ml LH (Fig. 6, *A* and *B*; SI Appendix, Fig. S4*F*). Both LH and FSK decreased (30–74% reduction) phosphorylation of ACACA at Ser79, a site-specific for enzyme inactivation (49), *via* inhibition of AMPK (21, 49). Treatment with a PKA inhibitor, H89 (10 μM), prevented LH-mediated effects on phosphorylation of ACLY and ACACA in luteal cells, confirming that phosphorylation of these proteins is mediated by LH/PKA signaling in luteal cells (SI Appendix, Fig. S5*F*).

Next, we tested if ACLY mediates an important function in energy production by luteal cells. Blockage of ACLY reduced basal and LH stimulated ATP and cAMP production (30% and 60% reduction) as well as the phosphorylation of PKA substrates. Inhibition of ACLY did not affect the content of steroidogenic machinery proteins such as CYP11A1 or steroidogenic acute regulatory protein (STAR), mitochondrial OXPHOS proteins and TOM20, or catalytic subunits of PKA α/β/γ (Fig. 6, *C*–*F*; SI Appendix, Fig. S6*A*). Knockdown or inhibition of CPT1A also inhibited LH-stimulated cAMP production (77% reduction) and phosphorylation of PKA substrates without changes in the content of catalytic subunits of PKA α/β/γ (Fig. 6, *G* and *H*; SI Appendix, Fig. S6, *B* and *C*). Similarly, pretreatment with ACLY or CPT1A inhibitors abrogated stimulatory effects of PKA activator, FSK on cAMP, phosphorylation of PKA substrates, and progesterone production without changes in content of STAR (SI Appendix, Fig. S6, *D*–*G*).

## Discussion

LH-driven alterations in the metabolism of luteal tissue have been recognized for more than 50 years (33, 41). However, these early studies referred only to changes in selected metabolites, such as glucose, lactate, pyruvate, or lipids in the granulosa cells, luteinized luteal tissue, or whole luteal tissue, without providing information on global metabolic changes induced by LH in highly steroidogenic luteal cells (33, 50, 51). By using complementary unbiased metabolic approaches, we identified prominent metabolic pathways acutely affected by LH in purified populations of highly steroidogenic primary small luteal cells. We also analyzed the flux of [U-^13^C_6_]-glucose and energetic pathways in LH-stimulated luteal cells. The use of inhibitors of selected metabolic routes revealed the relative importance of specific metabolic pathways such as glycolysis, TCA cycle, and lipogenesis in the acute response to LH, a vital hormone responsible for the formation, development, and maintenance of luteal function and proper steroidogenesis (52, 53, 54). These results provide novel evidence for LHCGR-stimulated metabolic pathways leading to lipid synthesis, which are of particular significance in steroidogenic cells as they serve to sustain energy homeostasis and regulate substrate availability for the production of prodigious amounts of steroid hormones. In addition, the present study demonstrates LHCGR-mediated posttranslational modifications of enzymes involved in *de novo* lipogenesis and the role of *de novo* fatty acid synthesis and fatty acid oxidation in energy homeostasis and LHCGR/PKA signaling and, consequently, progesterone production.

The findings in this study indicate that LH acutely enhances glucose uptake and glycolysis in the steroidogenic small luteal cells. Previous studies showed that gonadotropins stimulate glycolysis in the primary granulosa cells of sheep, cow, macaque, human, or mice (28, 29, 30, 31, 42, 55, 56, 57, 58) as well as mice or rats ovarian follicles cultured or grown *in vitro* (32, 42) or luteal tissue. In contrast to previous studies in the cow (59), inhibition of glucose uptake and blockage of hexokinase, an enzyme catalyzing conversion of glucose to glucose-6-phosphate, did not affect progesterone production by luteal cells. Since cellular metabolism is an adaptable and flexible network, inhibition of these steps of glycolysis could activate mechanisms overcoming the effects of enzyme inhibition. For example, a decrease in the supply of exogenous glucose could enhance the hydrolysis of storage carbohydrates such as maltotriose and maltotetraose or fructose, as observed in this study, but this remains to be experimentally tested. Caution is needed with this speculation because *in vivo* treatment with 2-deoxy-D-glucose, an inhibitor of hexokinase, prevented both the occurrence of the estrous cycle and the formation of the corpus luteum in cows (60). In contrast, blockage of PKM2, an enzyme that catalyzes the final step of aerobic glycolysis, *i.e.*, dephosphorylation of phosphoenolpyruvate to pyruvate with the simultaneous generation of ATP (36), abrogated LH-mediated stimulatory effects on steroidogenesis in luteal cells. Pyruvate can also be obtained from the sialic acid pathway and catabolism of glutamine, alanine, cysteine, serine, tryptophan, and threonine. As elevations in alanine and threonine were observed in response to LH, we speculate these amino acids are not the primary substrates to fuel the TCA cycle in luteal cells but may be instead used for anabolic processes (39, 61). Notably, inhibition of PKM2 reduced glycolysis, confirming the effective action of this inhibitor. Glycolysis supports cellular levels of NAD and NADH, cofactors required for proper oxidation occurring in mitochondria (61); therefore, we posit that LH enhances aerobic glycolysis, which supplies cells with cofactors and pyruvate required for the proper function of the TCA cycle and mitochondrial oxidation.

Untargeted metabolomic and fluxomic analysis revealed increases in the concentrations of pyrimidines and purines in LH-treated cells. There were also significant changes within the HBP, a branch of glycolysis related to producing UDP-glucose, an essential substrate for protein or lipid glycosylation. LH stimulated the activity of G6PD in rat or bovine luteal explants, and therefore, G6PD was identified as the most effective dehydrogenase in the luteal production of NADPH, a cofactor required for progesterone synthesis (62, 63). We did not measure enzyme activity; however, experimental conditions that blocked G6PD did not affect progesterone production despite high concentrations of inhibitor applied in the experiments. Thus, it seems reasonable to think that the PPP is not required for the regulation of basal or acute luteal progesterone production in response to LH and may rather serve a crucial role in the translation or synthesis of signaling molecules such as glycolipids, proteoglycans, or glycoproteins (37, 64, 65).

The mitochondrial TCA cycle yields cofactors for the function of the ETC and ATP production. The TCA cycle also provides precursors for various biosynthetic pathways, such as lipid or nucleotide synthesis, and supplies cells with NADPH (65). Inhibition of succinate dehydrogenase activity, an enzyme of the TCA cycle, stimulated G6PD activity and simultaneously led to the accumulation of cholesterol in the immature rat ovary (63). Because cholesterol is cleaved to pregnenolone by CYP11A1, which uses NADPH as a cofactor, the results of previous studies indicated ineffective NADPH output and impaired activity of CYP11A1 when the TCA cycle was interrupted. In the present study, untargeted metabolomics and fluxomics analysis with labeled [U-^13^C_6_]-glucose revealed significant LH-induced changes in TCA cycle metabolites. Pretreating luteal cells with CPI613, an inhibitor of pyruvate dehydrogenase and α-ketoglutarate dehydrogenase, blocked LH-stimulated progesterone synthesis, revealing a crucial role for the TCA cycle in steroidogenesis. As we did not see an effect of PPP inhibition on progesterone production, we posit that the TCA cycle constitutes a leading source of NADPH required for CYP11A1 activity and cofactors essential for maintaining the proper function of ETC in luteal cells.

Citrate produced within the TCA cycle can be transported to the cytoplasm and cleaved by ACLY to form acetyl-CoA and oxaloacetate. The product, acetyl-CoA, can be used to synthesize fatty acids or cholesterol as well as posttranslational modifications of proteins (26, 64). Acetyl-CoA can also be obtained from acetate by ACSS2 (37). Herein, LH rapidly depleted citrate and stimulated the incorporation of labeled-glucose carbons into acetate. Incorporation of [U-^14^C]-acetate into intraluteal fatty acids and progesterone was observed previously in rat luteal tissue following injection with LH (44). Recently, the importance of pantothenic acid, a precursor for CoA synthesis and formation of acetyl CoA, was identified in a metabolomic analysis MA-10 Leydig cells. The same study found that inhibition of pantothenic acid synthesis reduced hCG-stimulated progesterone production (66). However, this study did not evaluate the metabolic response to LH or cAMP in MA-10 cells. Herein, LH and Forskolin regulated the phosphorylation of ACLY and ACACA at sites required for enzyme activity. ACLY and ACACA are enzymes catalyzing the initial steps in lipogenesis and fatty acid synthesis (67, 68). Blockage of ACSS2 did not affect LH-promoted progesterone production by luteal cells. In contrast, inhibition of ACLY abrogated the stimulatory effects of LH on the production of progesterone, ATP, and cAMP and the phosphorylation of PKA substrates in luteal cells. These results emphasize the importance of ACLY and acetyl-CoA metabolism in LH-stimulated energy production, which is essential for the proper function of LHCGR/PKA signaling and the steroidogenic capacity of luteal cells.

Our findings show LH-mediated utilization of pantothenate, required for CoA formation, and incorporation of [U-^13^C_6_]-glucose-derived carbons into CoA, a cofactor essential for acetyl-CoA and lipid synthesis (38). Furthermore, LH stimulated the incorporation of [U-^13^C_6_]-glucose-derived carbons into carnitine. These findings suggest enhanced production and utilization of fatty acids synthesized from glucose. Fatty acids constitute an essential energy source and substrate for synthesizing different lipid mediators, including prostaglandins, leukotrienes, and steroid hormones (38). Fatty acids are transported to the mitochondria *via* the mitochondrial carnitine system, where they are oxidized (69). To determine whether fatty acids are utilized for energy and steroid synthesis, we interrupted activity of CPT1A, a transporter of fatty acids to the mitochondria. Either inhibition of CPT1A by using small molecule inhibitors or knockdown of *CPT1A* significantly blocked LH-stimulated progesterone production without changes in the content of steroidogenic machinery and mitochondrial proteins. Concurrent with the inhibition of CPT1 were decreases in the LH-promoted production of ATP, cAMP, and phosphorylation of PKA substrates. Thus, we propose that endogenous fatty acids are required for sufficient mitochondrial energy production to fuel LHCGR/PKA signaling in luteal cells. In the light of our previous findings, we speculate that LH/PKA/ACLY pathway maintains energy homeostasis in luteal cells and limits activation of AMP-activated kinase (AMPK), which inhibits LH-stimulated progesterone production *via* decreasing the activity of enzymes related to lipolysis and *de novo* fatty acids synthesis (23, 24).

Herein, we documented luteal cholesterol utilization reflected by a rapid increase in the concentration of isocaproate, the sidechain of cholesterol cleaved by the mitochondrial CYP11A1 enzyme, and the acute and sustained increase in progesterone production. The conditions for metabolic analysis precluded incubations with serum (which contains lipoproteins), thus removing extracellular cholesterol as a source of cholesterol for progesterone synthesis. We also observed a slight but significant decrease (28%) in total cellular cholesterol content after 240 min of incubation with LH. Those changes were accompanied by elevated intraluteal content of cholesterol precursors, *i.e.*, lanosterol and squalene, suggesting the induction of *de novo* cholesterol synthesis. Activation of *de novo* cholesterol synthesis has recently been reported in mouse ovaries during the LH surge, and blocking of SREBP disrupted steroid synthesis (70). In contrast, blocking HMGCR activity, a crucial enzyme in cholesterol synthesis, in fully differentiated luteal cells, did not affect LH-stimulated progesterone production. *De novo* cholesterol synthesis is an anabolic process, and although the enzymes involved in the cholesterol synthesis are abundant in the mature corpus luteum (71), *de novo* cholesterol synthesis does not appear to be rate limiting for progesterone synthesis in small luteal cells. Our findings support the notion that this pathway is not a leading source of cholesterol for steroidogenesis in luteal cells (72). This is further supported by our recent findings showing that in response to LH, a pool of cholesterol esters stored in lipid droplets of bovine small luteal cells (21, 23) is hydrolyzed by PKA-activated hormone-sensitive lipase (HSL, also known as LIPE). The released cholesterol is trafficked to the mitochondria for progesterone synthesis. A limitation of the current study is the employment of *in vitro* incubations of primary cells under serum-free conditions. However, inhibition of hormone-sensitive lipase-abrogated lipoprotein stimulated progesterone synthesis in the presence and absence of LH, indicating that the stored cholesterol esters are the hormone-sensitive reservoir of cholesterol and crucial for acute LH-stimulated progesterone synthesis (21). Similar observations were reported in adrenal and Leydig cells (73, 74). *In vivo* studies employing additional experimental models are needed to examine progesterone responses after employing selective small molecule inhibitors of the key metabolic processes identified in this report.

**Figure 7:**
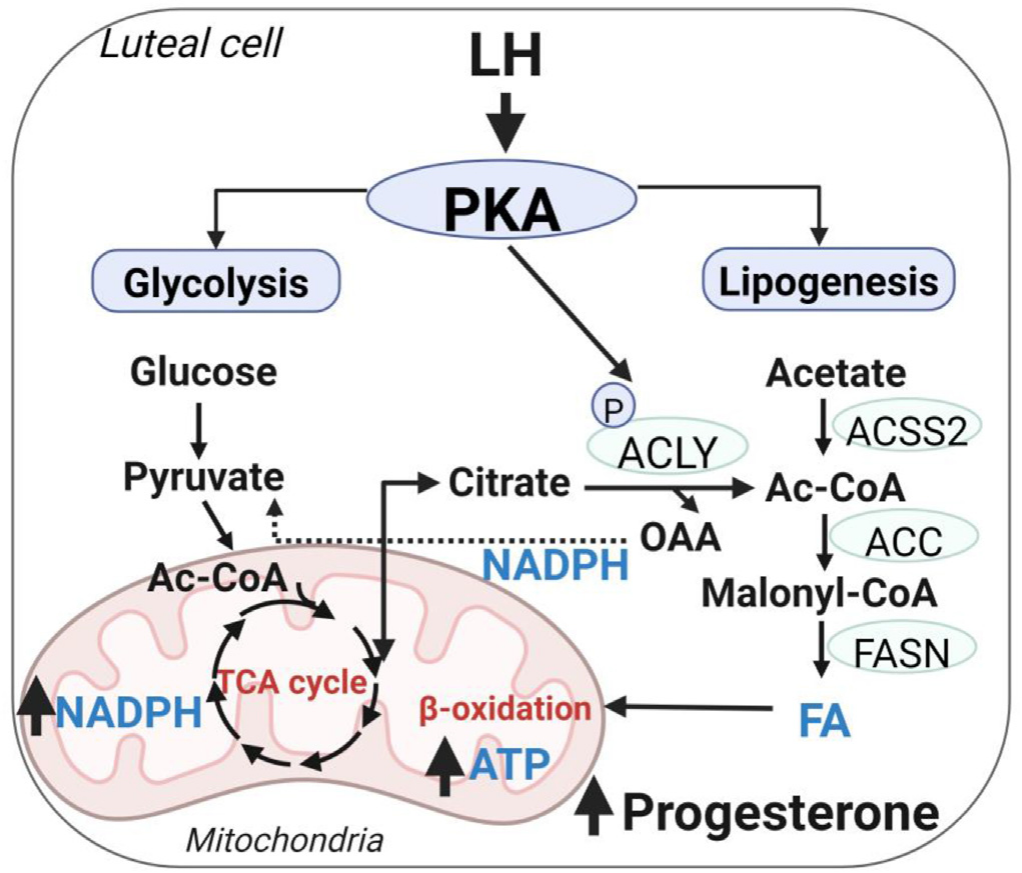
**Central role for glycolysis and fatty acids in LH-responsive progesterone synthesis.** LH *via* PKA activates metabolic pathways leading to production of acetyl-CoA (Ac-CoA) and *de novo* FA synthesis. Ac-CoA can be obtained from citrate or acetate *via* ACLY or ASCC2. FA can be used for β-oxidation and citrate can be used to produce pyruvate with simultaneous production of NADPH. Obtained energy is required for proper LHCGR/PKA signal transduction and progesterone production. ACLY, ATP citrate lyase; FA, fatt acid; LH, luteinizing hormone; PKA, protein kinase A.

The present study shows for the first time that LH, the main luteotrophic hormone in domestic animals and humans, evokes acute global metabolic changes that support progesterone production by highly steroidogenic luteal cells. LH stimulated changes in various branches of glucose metabolism, such as glycolysis and the TCA cycle, as well as induced posttranslational modifications of ACLY and ACACA, enzymes involved in *de novo* lipogenesis. These findings demonstrate that LH stimulates enzymes and metabolic pathways leading to the synthesis of *de novo* fatty acids, which are a primary source of energy, required for proper LHCGR/PKA signaling, and consequently progesterone production (Fig. 7). Since the LHCGR signaling pathway is conserved among different animal species and humans, these findings may have a translational impact.

Further mechanistic studies are required to explain the genomic and nongenomic action of LHCGR/PKA signaling on components of metabolic pathways in luteal cells. LH can simultaneously activate the phospholipase C/calcium/protein kinase C signaling pathway and this pathway is known to impact cellular glycolysis and the TCA cycle, making additional studies necessary to identify metabolic nodes shared by both pathways or alternative parallel metabolic pathways that contribute progesterone synthesis. Because this study evaluated the acute response to LH (up to 4 h), additional studies are necessary to determine the longer tropic responses to LH.

## Experimental procedures

### Reagents

Bovine LH was purchased from Tucker Endocrine Research Institute. Protease and phosphatase inhibitor cocktails were purchased from Sigma Chemical Co. M199 and fetal bovine serum (FBS) were obtained from Cambrex. Type II collagenase was obtained from Atlantic Biologicals. Forskolin were from EMD Chemicals, Inc. Mito Stress and Glycolysis kit were purchased from Agilent Technologies, Inc. H89, Shikonin, CPI613, BMS303141, 6AN, Fluvastatin, and Lonidamine were purchased from Tocris Bioscience. ACSS2 inhibitor was obtained from Selleck Chemicals LLC. Etomoxir (sodium salt) was obtained from Cayman Chemical. Progesterone assay was purchased from Diagnostic Systems Laboratories Inc. [U-^13^C_6_]-glucose was purchased from Sigma Chemical Co. Glucose uptake assay was obtained from Promega. Phospho-ACLY (Ser455; #4331), phospho-ACACA (Ser79; #3661), CYP11A1 (#14217), phospho-PKA substrates (#9624), and TOM20 (#42406) antibodies were purchased from Cell Signaling Technology, Inc. Total OXPHOS Antibody Cocktail (ab110413), STAR (ab96637), ATP assay, and L-lactate assay were purchased from Abcam. PKAα/β/γ subunits antibodies were obtained from Santa-Cruz Biotechnology Inc (sc-365615). siCPT1A (ON-TARGETplus Custom siRNA; cat no. CTM-3909; SMICK-000005; Dharmacon. Direct Cyclic AMP Elisa Kit was purchased from Arbor Assays. HRP linked anti-mouse (#115035205) or anti-rabbit (#111035144) antibodies were obtained from Jackson ImmunoResearch Laboratories Inc.

### Isolation and culture of bovine luteal cells

Isolation and culture of bovine luteal cells was performed as previously described (47). Bovine ovaries of early pregnant cows (fetal crown rump length < 12 cm) were collected in a local abattoir (XL Four Star Beef) and transported to the laboratory on ice in a cold M199 supplemented with antibiotics (100 U/ml penicillin-G-sodium, 100 μg/ml streptomycin sulfate, and 10 μg/ml gentamicin sulfate). The luteal tissue was dissociated with collagenase and purified luteal cells were prepared using centrifugal elutriation as previously described (75). This procedure removes endothelial cells and large luteal cells resulting in preparations of small luteal cells with a purity >92%. The cell viability as determined by the trypan blue exclusion test was >90%. For metabolomic experiments, small luteal cells were used as cell suspensions on the day of preparation. After treatment with control medium or LH, the luteal cells were rapidly pelleted and the media were collected and immediately frozen. The cell pellets were rinsed with cold PBS and repelleted. The cell pellet was snap frozen in liquid nitrogen prior to metabolomics analysis. For experiments with inhibitors, siRNA and Western blots, enriched bovine steroidogenic small luteal cells were cultured overnight on plates in basal medium (M199 containing 0.1% bovine serum albumin and antibiotics) with 5% FBS at 37 °C in a humidified atmosphere of 5% CO_2_. On the day of experiment, medium was removed and cells were washed with warm PBS. Fresh FBS-free medium was added and cells were equilibrated for 2 h before applying various treatments as detailed in the information included in the figure legends.

### Metabolic analysis

Sample preparation, metabolite extraction and metabolite detection were performed by the Metabolon company (Metabolon Inc) based on a global unbiased platform. Briefly, the samples were prepared by using Hamilton MicroLab STAR system and the resulting extract was divided and prepared by using TurboVap (Zymark) and vacuum drying machine for the following instrument: (i) ultra-Performance Liquid Chromatography-Tandem Mass Spectrometry (UPLC-MS/MS) with positive ion mode electrospray ionization (Waters Acquity UPLC and Thermo-Finnigan LTQ mass spectrometer; scan range, 80–1000 m/z); (ii) UPLC-MS/MS with negative ion mode electrospray ionization; (iii) liquid chromatography (LC) polar platform; iv) Gas Chromatography-Mass Spectroscopy (GC-MS, Thermo-Finnigan Trace DSQ fast-scanning single-quadrupole mass spectrometer; scan range, 50–750 m/z). Data were extracted using Metabolon's hardware and software (76) (https://www.metabolon.com/) constructed *via* Microsoft's NET technologies and compounds were identified based on Metabolon library recording *m/z*, retention time/index, and chromatographic data of more than 3300 molecules.

### NMR sample preparation and data collection for untargeted metabolomics

The dried metabolite extracts were reconstituted in 600 μl of 50 mM phosphate buffer in 99.8% D_2_O (Isotec) at pH 7.2 (uncorrected). 50 μM of TMSP(3-(tetramethysilane) propionic acid-2,2,3,3-d4) were added for spectral referencing. The solution was mixed by gentle vortexing and centrifuged at 13,000 rpm for 5 min, and then the supernatant was transferred to 5 mm NMR tube. The NMR data were collected using Bruker AVANCE III 700 MHz spectrometer equipped with 5 mm triple-resonance cryogenic probe (^1^H, ^13^C, and ^15^N) with a Z-axis gradient were used to acquire the NMR data. The sample collection was automated using a SampleJet sample changer, ATM (automatic tuning and matching), and Bruker IconNMR software (https://www.bruker.com/). The NMR experiment was conducted at 300 K with 32 K data points, 256 scans, 16 dummy scans, and a spectral width of 11,160 Hz using an excitation sculpting pulse sequence.

### NMR sample preparation and data collection for targeted metabolomics using stable isotope tracers

The dried metabolite extracts were reconstituted in 450 μl of 50 mM phosphate buffer in 99.8% D_2_O (Isotec, St Louis, MO) at pH 7.2 (uncorrected). 500 μM of TMSP(3-(tetramethysilane) propionic acid-2,2,3,3-d4) were added for spectral referencing. The solution was mixed by gentle vortexing and centrifuged at 13,000 rpm for 5 min, and then the supernatant was transferred to 5 mm NMR tube. The NMR data were collected using Bruker AVANCE III 700 MHz spectrometer equipped with 5 mm cryoprobe. The sample collection was automated using SampleJet and Bruker IconNMR software. A 2D ^1^H-^13^ C heteronuclear single quantum coherence spectra were collected at 300 K with 2 K data points in the direct dimension, and 256 data points in the indirect dimension for 24 scans, 16 dummy scans.

### NMR data analysis for untargeted and targeted metabolomics

MVAPACK software (http://bionmr.unl.edu/mvapack.php) was used to analyze the untargeted metabolomics NMR data (77). A principal component analysis model was generated using raw NMR data that was Fourier transformed, automatically phased, and binned using adaptive intelligent binning, normalized using standard normal variate normalization, and noise was removed as stated by (78, 79, 80, 81). Orthogonal projections to latent structures discriminant analysis scores and back-scaled loadings were generated using full intact spectrum. Spectra were normalized using standard normal variate normalization, noise was removed as stated earlier and scaled using Pareto scaling. The Human Metabolomics Database and Chenomx NMR Suite 7.6 (http://www.chenomx.com/) were used for metabolite identification (78, 79, 80, 81, 82). 2D ^1^H-^15^N heteronuclear single quantum coherence spectra were processed using NMRPipe, and the time series was analyzed using NMRviewJ to obtain peak intensities over the course of the experiment, as previously explained.

### Seahorse analysis

#### Mito stress assay

Mitochondrial OCR was measured using a Seahorse XF^e^96 analyzer (Seahorse Bioscience). In brief, primary luteal cells were plated on XF^e^96 cell culture microplate (40 × 10^4^ cells per well) and incubated overnight. Next day, cells were washed with warm PBS and medium was replaced with fresh one. Cells were pretreated for 60 min with inhibitors, either Etomoxir (30 μM) or UK5099 (1 μM). Then, culture medium was changed with assay medium (base medium with L-glutamine, glucose, and pyruvate) and cells were incubated with LH (10 ng/ml) for 60 min in non-CO_2_ incubator. The assay was done following injections: oligomycin (2 μM), FCCP (0.5 μM), and rotenone (0.5 μM) combined with antimycin (AA; 0.5 μM). Before and after each injection was performed three measurements of OCR and ECAR for each 3 min. First, the basal oxygen consumption rate (basal respiration) was measured. Afterward, oligomycin, which inhibits ATP synthase activity, was injected to reveal ATP production coupled with mitochondria respiration. Next, FCCP that increases proton pump was injected and allowed maximal mitochondrial respiration. Finally, rotenone combined with antimycin A was injected to inhibit the flux of electrons through complexes I and III; so, the remaining oxygen consumption rate was primarily due to nonmitochondrial respiration. The spare respiratory capacity was calculated by subtracting the basal respiration from the maximal respiration. At least four technical replicates were used for each treatment, and the experiments were repeated three times to confirm the results.

#### Glycolysis stress assay

The Glycolysis Stress Kit was applied to determine glycolysis in luteal cells. Similarly, primary luteal cells were plated on XF^e^96 cell culture microplate (40 × 10^4^ cells per well) and incubated overnight. Next day, cells were washed with warm PBS and medium was replaced with fresh one. Before assay, cells were pretreated with Shikonin (5 μM) in culture medium. Afterward, cultured medium was replaced with assay medium (base medium supplemented with 2 mM L-glutamine) and treated with LH (10 ng/ml) in non-CO_2_ incubator. The following injections were performed during the assay: glucose (10 mM), oligomycin (2 μM), and 2-DG (5 mM). Before and after each injection, OCR and ECAR were measured three times every 3 minutes. Four technical and biological replicates were used for each treatment group.

### Lactate concentration assay

To confirm glycolysis, media samples were collected after seahorse analysis to determine lactate concentration using the L-lactate assay according to manufacturer’s protocol (Abcam). Absorbance values at 450 nm were obtained using a plate reader. Readings were corrected for background absorbance using the absorbance value from the assay medium only, and the corrected values were applied to a standard curve to calculate extracellular lactate levels.

### Glucose uptake assay

Small luteal cells (10,000 per well) were plated on 96-well plates and cultured overnight in M199 supplemented with antibiotics and 5% FBS. Next day, media were removed, cells were washed with PBS and fresh media without FBS was added. After 2 hours of equilibration, cells were treated with LH (10 ng/ml) for 10 and 60 min. Afterward, media were removed, cells were washed with PBS and 1 mM 2-deoxy-D-glucose was added. After 10 min of incubation at room temperature, the stop buffer followed by the neutralization buffer was added and the cells were briefly shaken. Then, 2-deoxy-D-glucsoe-6-phopshate detection reagent was added, and cells were incubated for 45 min at room temperature. Luminescence was recorded using luminometer FLUOstar Optima (BMG Labtech).

### ATP assay

Luteal cells were plated on 96-well plates with clear bottom and cultured overnight in M199 supplemented with antibiotics and 5% FBS. Next day, media was removed, cells were washed with PBS and fresh media without FBS was added. After 2 hours of equilibration, cells were pretreated with inhibitors BMS303141 (10–50 μm) or Teglicar (10–50 μM) for 60 min and then treated with LH (10 ng/ml) for 240 min. Afterward, ATP assay was performed according to manufacturer’s protocol. Cells were lysed for 5 min by using detergent solution supplied in the assay, and then substrate solution was added, and cells were incubated for 5 min, adapted in dark for 10 min and luminescence was measured by using luminometer FLUOstar Optima (BMG Labtech).

### cAMP assay

cAMP concentration was measured in media samples collected after incubation of luteal cells with inhibitor and/or LH. Standards and media samples were pipetted onto 96-well coated with an antibody to capture sheep immunoglobulin G following primer plate. Next, a cAMP-peroxidase conjugate was added to both standards and samples. After 2 h incubation, plate was washed, and substrate was added. After short incubation, the reaction was stopped, and the intensity of the generated color was detected in a microtiter plate reader at 450 nm. Data were analyzed by using online tool provided by manufacturer at MyAssays website (www.myassays.com/arbor-assays-cyclic-amp-direct-eia-kit-non-acetyl.assay).

### Western blot analysis

After *in vitro* experiments cells were collected and lysed for Western blot analysis as previously described (47). Lysates were subjected to separation on 10% SDS-PAGE and transferred onto nitrocellulose membranes, which were then blocked with 5% bovine serum albumin in Tris-buffered saline with 0.1% Tween-20 (TBST) at room temperature for 1 h. Then, membranes were incubated with primary antibodies at 4 °C overnight. Next day, membranes were washed three times with TBST and blocked in secondary anti-mouse or anti-rabbit HRP-conjugated antibodies (1: 10,000) in 5% nonfat milk in TBST for 1 h, followed by a second series of three washes in TBST. Blots were imaged and quantified using iBright CL1500 Imaging System (Thermo Fisher Scientific Inc).

### siRNA transfection

*CPT1A* was knocked down using specific CPT1A silencing RNA (siRNA) to determine the role of CPT1A on luteal progesterone production, cAMP production, PKA signaling and steroidogenic machinery, or mitochondrial proteins. Small luteal cell populations were transfected with Lipofectamine RNAimax and control siRNA (siCTL) or si*CPT1A* (ON-TARGETplus Custom siRNA (CTM-385778; HOUSF-000005) in opti-MEM culture medium. After 6 h, 5% FBS was added to culture media and cells were maintained for 48 h. Knockdown of *CPT1A* was confirmed by Western blotting. Following transfection, luteal cells were treated with LH (10 ng/ml) for 4 h. Media were collected for progesterone concentration analysis and cell lysates were immediately collected for further Western blotting analysis.

### Progesterone ELISA

Luteal cells were cultured in 48-well plates. Media samples were collected to determine progesterone concentration using ELISA method (Diagnostic Systems Laboratories, Inc) according to the manufacturer's instructions.

### Statistical analysis

Statistical analyses were conducted using GraphPad Prism v. 8.0 software (GraphPad Software, Inc., San Diego, CA, USA) (www.graphpad.com). All experiments were performed at least three times using luteal cells obtained from different animals. The data are presented as the means ± SD of the averages from multiple experiments. Data were analyzed by *t* test (unpaired), one- or two-way ANOVA, followed by Bonferroni multiple comparison tests. Statistical differences were considered as significant at *p* < 0.05.

## Data availability

All data are available in the main text or the supplementary materials.

## Supporting information

This article contains supporting information.

## Conflict of interest

The authors declare that they have no conflicts of interest with the contents of this article.
