# Supplementary material for: Metabolic control of luteinizing hormone-responsive ovarian steroidogenesis
Source: J Biol Chem. 2024 Nov 29;301(1):108042. doi: 10.1016/j.jbc.2024.108042 (PMC11732475; doi:10.1016/j.jbc.2024.108042)
Supplement: Supporting information [file mmc3.pdf]

Supporting Information For

Metabolic control of Luteinizing Hormone-responsive ovarian steroidogenesis

Emilia Przygrodzka *et al.*

*Corresponding author:

Emilia Przygrodzka, emiliap@umd.edu
John S. Davis jsdavis@unmc.edu

This PDF file includes:

Figs. S1 to S6

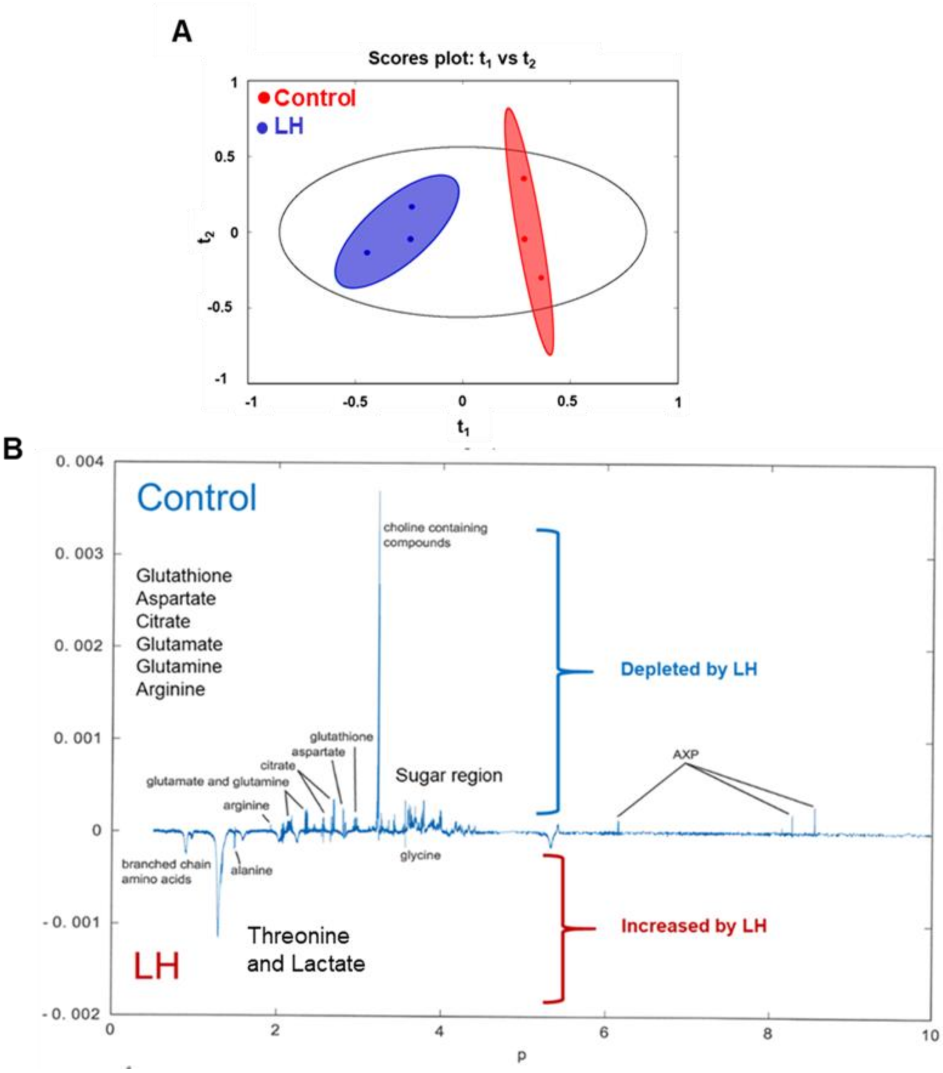

Fig. S1.
Enriched preparation of small luteal cells treated with LH (10 ng/ml) for 240 min were analyzed by Nuclear Magnetic Resonance (NMR) spectroscopy.
(**A**) Principal Component Analysis (PCA) plot showing grouping of sample preparation (n=3 per group) from untreated (Control; red circle) and LH-treated cells (blue circle).
(**B**) NMR spectrum showing the most significantly (depleted or increased) changed metabolites post-LH treatment.

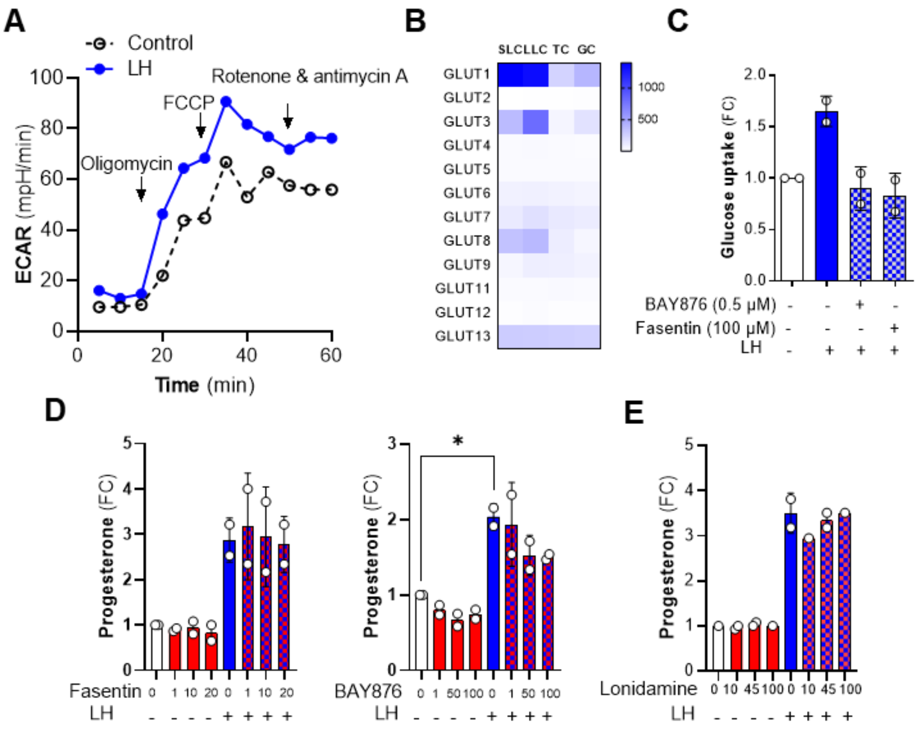

Fig. S2.

(**A**) Representative graph showing changes in the extracellular acidification rate (ECAR) in untreated (Control) and LH-treated cells (60 min) based on Seahorse Cell Mito Stress Test.

(**B**) Heatmap representing expression of glucose transporters (*GLUT 1-9*; *GLUT 11-13*) in the small and large luteal cells (SLC and LLC) as well as theca and granulosa cells (TC and GC). Data were obtained from previously performed microarray analysis (GSE83524).

(**C**) Glucose uptake measured by using luminescence method in the small luteal cells pretreated with inhibitors of GLUT1 (BAY876; 1 µM) or GLUT1/4 (Fasentin; 100 µM) and then treated with LH (10 ng/ml) for 60 min. Data are presented as a fold change (FC) and mean± SD (n=2).

(**D-E**) Progesterone production by the small luteal cells pretreated with inhibitor of Glut1 (BAY876; 1-100 µM) or Glut1/4 (Fasentin; 1-20 µM) or hexokinase (Lonidamine; 10-100 µM) and then treated with LH (10 ng/ml) for 240 min. Data are presented as a fold change (FC) and mean± SD(n=2).

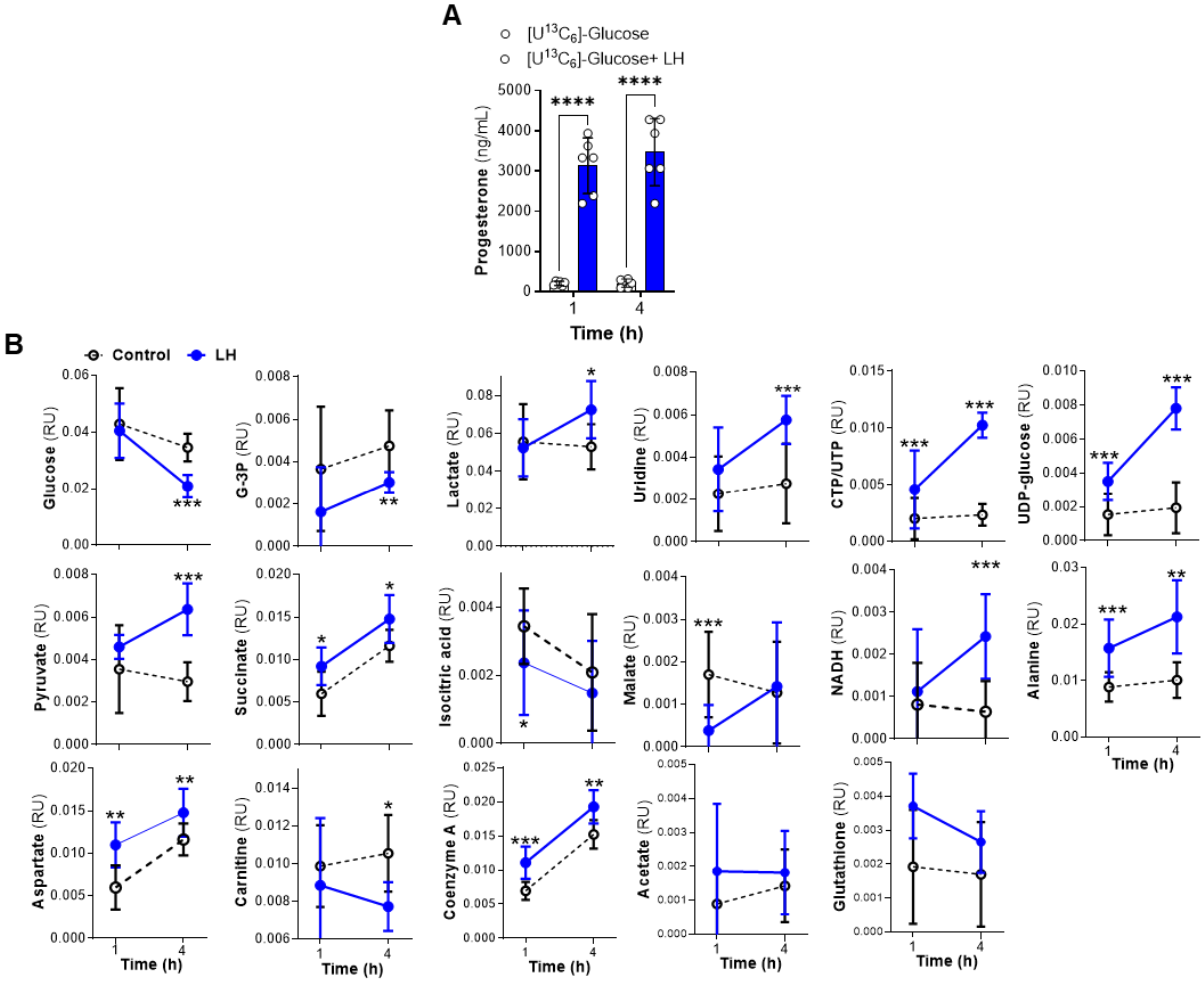

Fig. S3.

(**A**) Progesterone production by the small luteal cells treated with [U13C6]-labeled glucose (5 mM) alone or in the presence of LH (10 ng/ml) for 60- and 240-min. Data are represented as mean± SD (n=6) and were analyzed using one-way ANOVA test. Asterisks **** mean significant change with P < 0.0001.

(**B**) Time-dependent changes in the concentration of selected metabolites in small luteal cells cultured in medium with [U-13C6]-labeled glucose in the presence or absence (Control; black dotted line) of LH (blue solid line). Data are presented as relative units (RU) and mean± SD (n=6). Asterisks *, **, *** mean significant change with P < 0.05, P < 0.01, and P < 0.001, respectively. (n=6). Asterisks *, **, *** mean significant change with P < 0.05, P < 0.01, and P < 0.001.

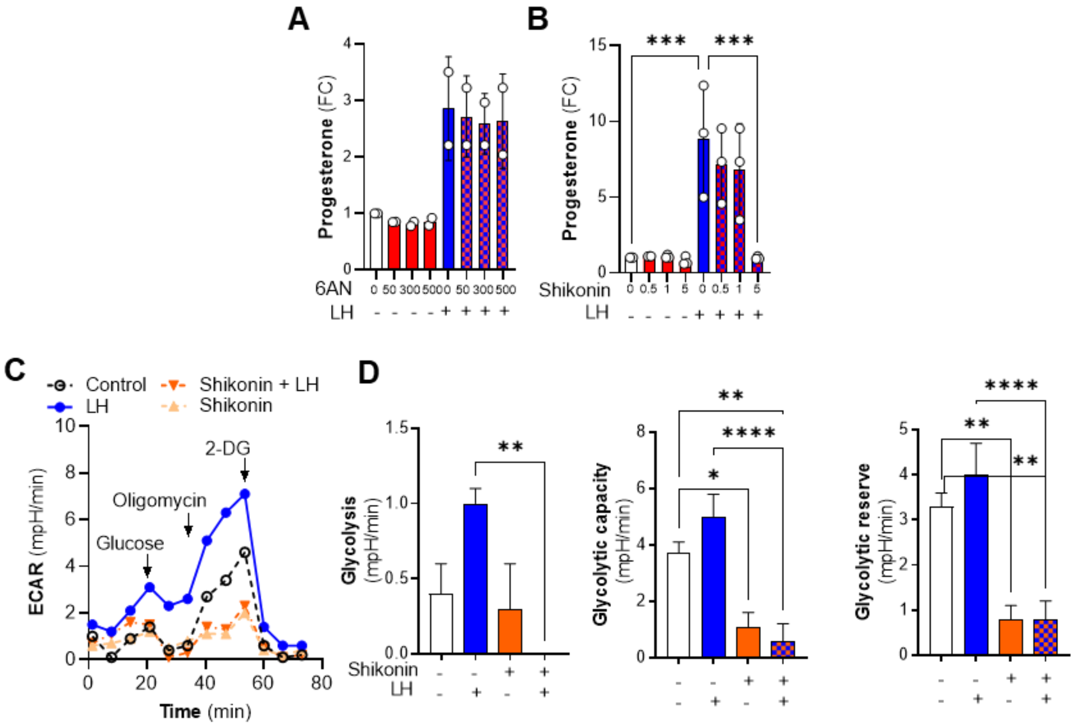

Fig. S4.

(**A-B**) Progesterone production by cells pretreated with inhibitor of glucose-6-phosphate dehydrogenase (G6PD; 6AN; 50-500 μM) or pyruvate kinase (Shikonin; 0.5-5 μM) and then treated with LH (10 ng/ml) for 240-min. Data represented as fold change (FC) and mean ± SD (n=3) were analyzed using one-way ANOVA test. Asterisks *** mean significant change with $P < 0.001$.

(**C**) Glycolytic rate was analyzed using Seahorse Glycolytic Rate Assay. Representative graph showing extracellular acidification rate (ECAR) in the small luteal cells pretreated with pyruvate kinase inhibitor (Shikonin) and then treated with LH (10 ng/ml) for 60 min.

(**D**) Glycolysis, glycolytic capacity, and glycolytic reserve in the small luteal cells pretreated with pyruvate kinase inhibitor (Shikonin) and then treated with LH. Data are represented as mean ± SEM (n=10-12) and were analyzed using a one-way ANOVA test. Asterisks *, ** and **** indicate significant change with $P < 0.05$, $P< 0.01$ and $P < 0.0001$, respectively.

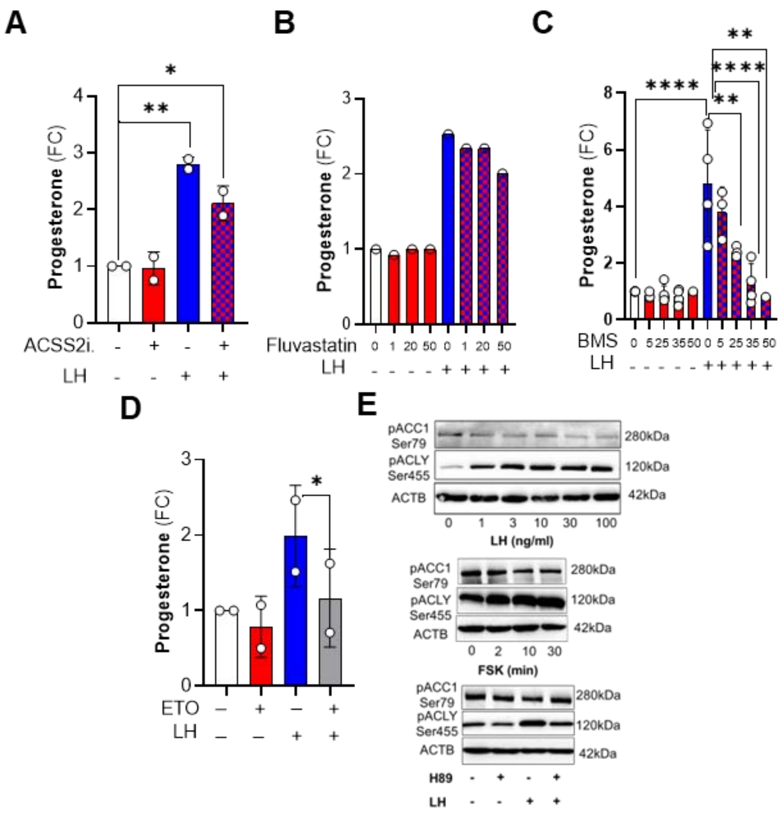

Fig. S5.
(**A-D**) Progesterone production by the small luteal cells pretreated with inhibitor of ACSS2 (ACSS2 i.; 10 µM), HMGCR (Fluvastatin; 1-50 µM), ACLY (BMS303141; 5-50 µM) or Etomoxir for 60 min and then treated with LH (10 ng/ml) for 240 min. Data are presented as a fold change (FC) and mean± SD (n=1-4). Data were analyzed using a one-way ANOVA test. Asterisks *, ** and **** mean significant change with $P < 0.05$, $P < 0.01$ and $P < 0.0001$, respectively.
(**E**) Representative blots showing phosphorylation of ACLY Ser455 and ACACA Ser89 in the small luteal cells treated with LH (1-100 ng/ml) for 30 min or cAMP/PKA activator forskolin (FSK; 10 µM) for 2-30 min as well as cells pretreated with PKA inhibitor (H89; 20 µM) and then treated with LH (10 ng/ml) for 10 min.

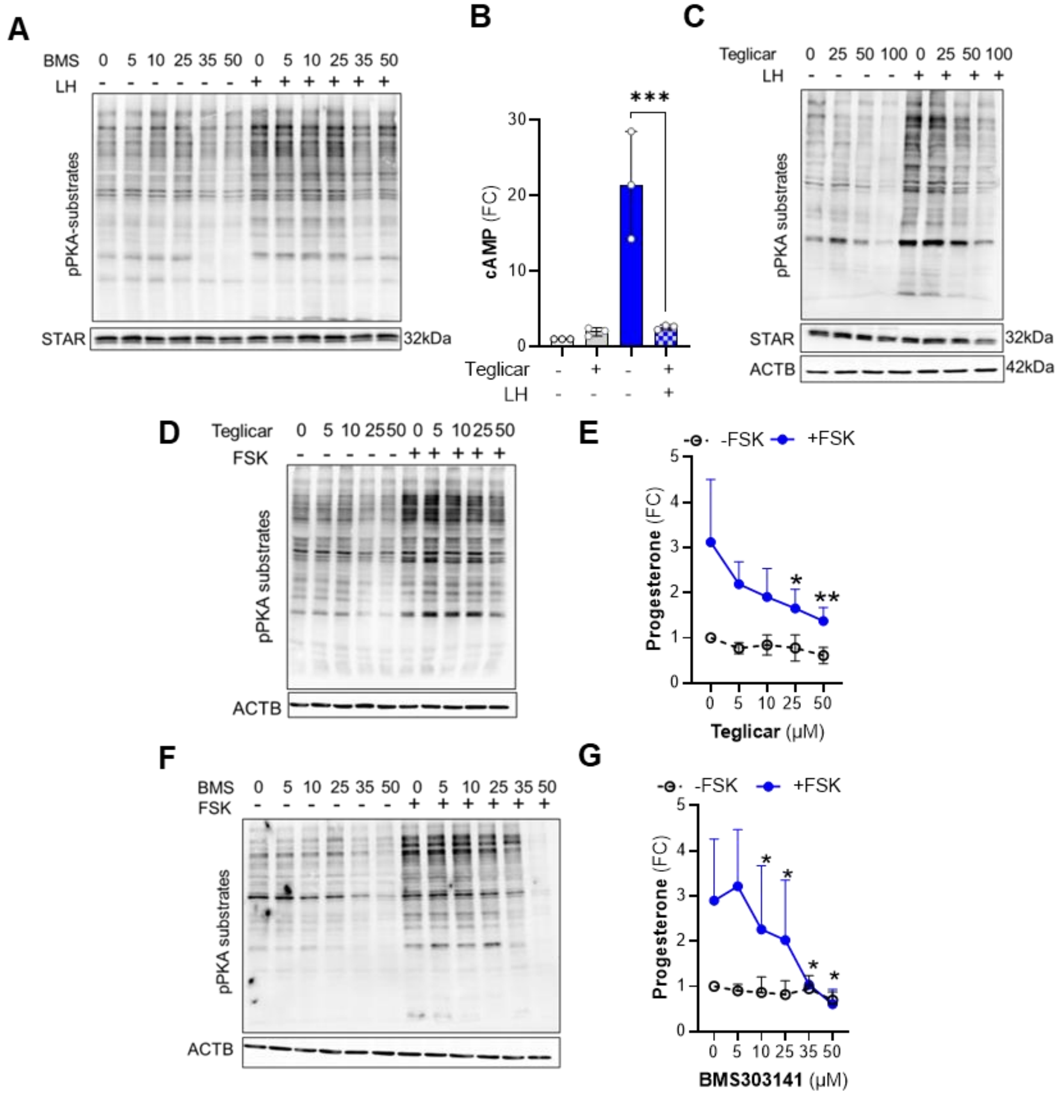

Figure S6.

(**A**) Representative blots showing phosphorylation of PKA substrates and content of STAR in the small luteal cells pretreated with ACLY inhibitor (BMS303141; 5-50 µm) and then treated with LH (10 ng/ml) for 240 min.

(**B**) cyclic AMP (cAMP) production by small luteal cells pretreated with CPT1A inhibitor (Teglicar; 25 µM) and then treated with LH (10 ng/ml) for 240 min. Data are presented as a fold change (FC) and mean± SD (n=2-3). Data were analyzed using a one-way ANOVA test. Asterisks ** mean significant change with $P < 0.01$.

(**C-D**) Representative blots showing phosphorylation of PKA substrates and content of STAR in the small luteal cells pretreated with CPT1A inhibitor (Teglicar; 5-50 µm) and then treated with LH (10 ng/ml) or PKA activator Forskolin (FSK; 10 µM) for 240 min.

(**E**) Progesterone production by small luteal cells pretreated with Teglicar (5-50 µM) and then treated with PKA activator Forskolin (FSK; 10 µM) for 240 min.

(**F-G**) Small luteal cells were pretreated with inhibitor of ACLY (BMS303141; 5-50 µM) and then treated with PKA activator forskolin (FSK; 10 µM) for 240 min. Representative blots of phosphorylation of PKA substrates in the small luteal cells pretreated with ACLY inhibitor (BMS303141) and then treated with forskolin (FSK).

(**G**) Progesterone production by the small luteal cells pretreated with ACLY inhibitor (BMS303141) and then treated with PKA activator forskolin (FSK). Data are presented as a fold change (FC) and mean±SEM (n=2-5). Data were analyzed using a two-way ANOVA test. Asterisks *, $P < 0.05$.
